# Biological Control of Chili Damping-Off Disease, Caused by *Pythium myriotylum*

**DOI:** 10.3389/fmicb.2021.587431

**Published:** 2021-05-13

**Authors:** Sajjad Hyder, Amjad Shahzad Gondal, Zarrin Fatima Rizvi, Rashida Atiq, Muhammad Irtaza Sajjad Haider, Nida Fatima, Muhammad Inam-ul-Haq

**Affiliations:** ^1^Department of Botany, Government College Women University, Sialkot, Pakistan; ^2^Department of Plant Pathology, Bahauddin Zakariya University, Multan, Pakistan; ^3^Department of Soil Science and SWC, PMAS Arid Agriculture University, Rawalpindi, Pakistan; ^4^Department of Plant Pathology, PMAS Arid Agriculture University, Rawalpindi, Pakistan

**Keywords:** antagonism, *Bacillus* spp., PGPR, *Pseudomonas* spp., plant growth promotion, *Pythium myriotylum*, IAA production, siderophore production

## Abstract

*Pythium myriotylum* is a notorious soil-borne oomycete that causes post-emergence damping-off in chili pepper. Of various disease management strategies, utilization of plant growth promoting rhizobacteria (PGPR) in disease suppression and plant growth promotion is an interesting strategy. The present study was performed to isolate and characterize PGPR indigenous to the chili rhizosphere in Pakistan, and to test the potential to suppress the damping-off and plant growth promotion in chili. Out of a total of 28 antagonists, eight bacterial isolates (4a2, JHL-8, JHL-12, 1C2, RH-24, 1D, 5C, and RH-87) significantly suppressed the colony growth of *P. myriotylum* in a dual culture experiment. All the tested bacterial isolates were characterized for biochemical attributes, and 16S rRNA sequence based phylogenetic analysis identified these isolates as *Flavobacterium* spp., *Bacillus megaterium*, *Pseudomonas putida*, *Bacillus cereus*, and *Pseudomonas libanensis*. All the tested bacterial isolates showed positive test results for ammonia production, starch hydrolase (except 4a2), and hydrogen cyanide production (except 4a2 and 1D). All the tested antagonists produced indole-3-acetic acid (13.4–39.0 μg mL^–1^), solubilized inorganic phosphate (75–103 μg mL^–1^), and produced siderophores (17.1–23.7%) *in vitro*. All the tested bacterial isolates showed varying levels of susceptibility and resistance response against different antibiotics and all these bacterial isolates were found to be non-pathogenic to chili seeds and notably enhanced percentage seed germination, plumule, redical length, and vigor index over un-inoculated control. Additionally, under pathogen pressure, bacterization increased the defense related enzymes such as Peroxidase (PO), polyphenol oxidase (PPO), and phenylalanine ammonia-lyase (PAL) activates. Moreover, the treatment of chili seeds with these bacterial isolates significantly suppressed the damping-off caused by *P. myriotylum* and improved PGP traits compared to the control. In addition, a positive correlation was noticed between shoot, root length, and dry shoot and root weight, and there was a negative correlation between dry shoot, root weight, and seedling percentage mortality. These results showed that native PGPR possesses multiple traits beneficial to the chili plants and can be used to develop eco-friendly and effective seed treatment formulation as an alternative to synthetic chemical fungicides.

## Introduction

Chili pepper (*Capsicum annuum* L.) is a member of *Solanaceae* family. It is an important vegetable crop worldwide, and is cultivated in Asia on large scale (Tariq et al., 2014). Chili accounts for almost 20% of the total vegetable growing area in Pakistan. It is consumed as a fresh or processed spice, and serves as a good source of vitamins A and C, phenolics, and carotenoids. Capsaicinoid compound derived from chili has many ethnopharmacological applications including anticancer, anti-obesity treatment, temperature regulation, pain therapy, and antioxidant effects (Meghvansi et al., 2010). Chili crop is vulnerable to more than 100 different types of pathogens during its various growth stages (Jayapala et al., 2019). Of the different microbial diseases that affect this plant, the damping-off and root rot disease caused by *Pythium myriotylum Drechsler.* is the most devastating disease in terms of seedling mortality at very early growth stages in nurseries, affecting the seedlings when they are in the cotyledonous stage. *Pythium* spp. are disease causative fungal-like organisms that result in 90% plant death as pre and/or post-emergence damping-off under favorable conditions. A study has shown that damping-off may affect from 5 to 80% of the seedlings and result in huge economic losses for farmers (Lamichhane et al., 2017). This disease is characterized by the typical symptoms of rotten roots, necrosis, wilt, water soaking lesions, and the decay of young seedlings (Horst, 2013). *P. myriotylum* is one of the most commonly occurring species in greenhouses, and in warm and moist soil and present wide host range (Ben-Yephet and Nelson, 1999). Watery soaked, sunken lesions can be seen on the stem at soil level or below the soil on roots, causing the seedling to fall over the ground (Smith, 1975) and excessive soil moisture leads to the development and movement of zoospores which attack the host plants.

Of various other practices, chemical seed coating is widely adopted in agriculture to control the disease (Dorrance et al., 2009; Rothrock et al., 2012; Kandel et al., 2016). Chemicals such as bleach, hydrogen peroxide, ethanol, and fungicides are extensively used to kill the pathogen inoculum present on seed coats (Mancini and Romanazzi, 2014). Chemical seed treatment is an effective practice in controlling the soil and seed borne pathogens but can pose a detrimental effect on seed germination and cause phytotoxicity (du Toit, 2004). Besides this, pesticide residues in soil and water are a potential threat to humans and the environment (Ouyang et al., 2016; Lamichhane et al., 2017), and many of these chemicals have been declared carcinogen pollutants in many countries (Bressa et al., 1997). Non-judicial use of many of these synthetic pesticides and fungicides has come under increasing public scrutiny in different countries (Bourguet and Guillemaud, 2016) and reports on pest resistance development are also increasing the threat for farming (Onstad, 2013). Furthermore, these fungicides are noxious to the survival of beneficial rhizosphere microbes (Hussain et al., 2009). Thus, there is a strong need to find cost effective and environmentally safe alternatives that can minimize or eliminate dependency on synthetic pesticides.

Plant growth promoting rhizobacteria are free-living or plant root colonized bacteria that confer plant growth promotion (Glick, 2012) and do not cause any harm to their hosts (Ryan et al., 2008). Bacteria belonging to *Pseudomonas*, *Azospirillum*, *Azotobacter*, *Klebsiella*, *Enterobacter*, *Alcaligenes*, *Arthobacter*, *Burkholderia*, *Bacillus*, and *Serratia* spp. improve plant growth (Kloepper et al., 1989; Souza et al., 2015) are used as biocontrol agents (Liu et al., 2007; Chen et al., 2009; Labuschagne et al., 2010; El-Sayed et al., 2014) and biofertilizers (Vessey, 2003). PGPR promote plant growth by different mechanisms which include the production of Indole acetic acid (IAA) (Etesami et al., 2015), phosphate solubilization (Panhwar et al., 2014), atmospheric nitrogen fixation (Kuan et al., 2016), ACC deaminase activity (Chen et al., 2013) and zinc solubilization (Gupta et al., 2015). PGPR suppress plant pathogens by employing various mechanisms such as competition, siderophores production, antagonism and induced systemic resistance (Gómez-Lama Cabanás et al., 2014), which activate multiple defense-related enzymes to challenge them against a broad spectrum of phytopathogens (Cazorla et al., 2007; Vanitha and Umesha, 2011). Peroxidases (PO) have been involved in many defense-related mechanisms, including the hypersensitive reaction, lignification, cross-linking of phenolics and glycoproteins, and the production of suberization and phytoalexin (Wojtaszek, 1997). Polyphenol oxidase (PPO) catalyzes the oxidation of phenolics to free radicals that react with biological molecules, thus hindering the pathogen development (Jockusch, 1966). Phenylalanine ammonia-lyase (PAL) plays an important role in the regulation of phenylpropanoid production (Achnine et al., 2004) and synthesis of various defense-related secondary compounds such as phenols and lignin (Tahsili et al., 2014).

Biological control is an alternative strategy to reduce the dependency on agro-chemicals in crop disease management programs (Postma et al., 2003) and the use of PGPR in disease management is helpful to reduce the detrimental effects of agro-chemicals on the environment. Many reports are available on the biocontrol potential of PGPR against *Pythium* spp. and plant growth promotion effect on tomato (Al-Hussini et al., 2019), potato (Kenawy et al., 2019), cucumber (El-Tarabily et al., 2009), sugar beet (Williams and Asher, 1996), cereals (Labuschagne et al., 2010), and many other major crops. In many cases, it has been observed that imported bioformulations sometimes fail to act up to their maximum potential due to climate change (Compant et al., 2010), nutrient availability (Kandeler et al., 2006), and the rhizosphere competence of the microbes (Lugtenberg and Kamilova, 2009). Thus, the identification and characterization of PGPR indigenous to chili rhizospheres is important to screen bacterial isolates that can suppress *P. myriotylum* inoculum and enhance chili growth in nurseries and greenhouses.

Considering the importance of chili production in an eco-friendly environment, this study aimed to isolate and screen native rhizobacteria for their biocontrol potential against the most virulent strain of *P. myriotylum in vitro*, to characterize bacterial agents based on morphological characters, and also by 16S rRNA sequence analysis, to examine the effect of bacterial treatment on seed germination, and to study the ability of PGPR to suppress *P. myriotylum* –induced damping-off and PGP effects on chili in pot experiments under growth room conditions. To our knowledge, this is the first report on native PGPR suppressing *P. myriotylum* and enhancing growth promotion in chili from Pakistan.

## Materials and Methods

### Pathogen Inoculum

Strains of *Pythium myriotylum* D. (PMyr-1 and PMyr-2) were previously reported as the causal agent of damping-off and root rot in chili pepper (*Capsicum annum* L.) from Punjab, Pakistan (Hyder et al., 2018). *Pythium myriotylum* was identified on morphological and molecular basis. The ITS1 and ITS2 rDNA sequences of these two virulent strains had been submitted in the GenBank database (accessions no. MF143429 and MF143430), which displayed a 99% identity with of *P. myriotylum* (accession no. HQ643704).

### Sampling and Isolation of Bacterial Isolates

Major chili growing fields in Rawalpindi (33.5651° N, 73.0169° E) Punjab, Pakistan were surveyed and rhizospheric soil samples strictly adhering to chili plant roots were taken from 15 to 20 cm depth along with the plant roots. All the soil samples were immediately processed for the isolation of rhizobacteria after reaching the laboratory. Bacteria were isolated from 10 g of soil samples by serial dilution plating on nutrient agar (NA) (HiMedia Laboratories) medium containing Petri plates (Joseph et al., 2012). For the isolation of root colonizing bacteria, 1 g of the root samples were washed with tap water, surface sterilized using 70% ethanol for 5 min, followed by 1% sodium hypochlorite (NaOCl) for 2 min, and then washed five times with sterilized distilled water (Kuan et al., 2016). Sterilized roots were crushed in distilled water aseptically with sterilized mortar and pestle, and were streaked on NA medium followed by incubation at 26 ± 2°C for 24–48 h. Morphologically discrete bacterial colonies were picked aseptically using a sterilized loop and sub-cultured on NA medium containing Petri plates. Bacterial isolates were stored at −80°C in equal volumes of nutrient broth (NB) medium and 30% glycerol for further use in experiments.

### *In vitro* Screening of Bacterial Isolates Against *Pythium myriotylum*

The rhizobacterial isolates (*n* = 110) were tested in repeated experiments for antagonistic potential against two virulent strains of *P. myriotylum* (PMyr-1 and PMyr-2) by using a dual culture technique (Rabindran and Vidhyasekaran, 1996) on PDA medium containing Petri plates. Small disks of actively growing *P. myriotylum* (5 mm) were placed in the middle of 9 cm Petri plates, and counter streaked on two sides by each rhizobacterial isolate about 2.5 cm from the fungal disks. The control plates contained fungal plugs without bacterial streaks. Petri plates were incubated at 26 ± 2°C, and inhibition zones (cm) were measured against each isolate 48 and 96 h after incubation. Each of the bacterial isolates were tested in five repeats to confirm the results. The percentage mycelial growth inhibition was recorded using the following formula:

Mycelialgrowthinhibition(%)=(R–rR)X 100

R is the radius of fungal mycelial growth in the control;

r is the radius of fungal mycelial growth in the treatment.

### Biochemical Featuring of Rhizobacterial Isolates

Bacterial isolates displaying consistent antagonistic responses in repeated dual culture tests were characterized based on Gram type reaction and fluorescence emission using the standard methods as described earlier (Cappuccino and Sherman, 2005). Potassium hydroxide (KOH) solubility tests were performed using the protocol as previously described by Kirsop and Doyle (1991). In this test, a 24-h old bacterial colony grown on NA medium was mixed thoroughly with 3% KOH solution on a glass slide and mixed thoroughly. The formation of mucoid thread confirmed the positive results for the bacterial isolates. Catalase tests were performed in accordance with the method described by Hayward (1960). Freshly grown bacterial culture on NA medium was mixed with one drop of 3% H_2_O_2_ on a glass slide. Rapid gas bubbles formation confirmed the positive test results. Levan production was tested using the procedure described by Lelliott and Stead (1987).

A carbohydrate fermentation test was performed in accordance with the procedure previously described by Aneja (2001). In this test, overnight grown bacterial cultures were inoculated in screw-capped tubes containing sterilized phenol red carbohydrate fermentation broth (1 g Trypticase; 0.5 g Sodium Chloride; 0.02 mg Phenol red and 0.5 g carbohydrate in 100 mL of distilled water). A change in medium color from red to yellow indicated the positive test results.

A hydrogen sulfide (H_2_S) production test was performed following the protocol described by Warren et al. (2005). Briefly, the 24 h old bacterial cultures grown on NB medium were aseptically inoculated on Sulfide indole motility (SIM) medium (HiMedia Laboratories, India) containing tubes followed by incubation at 37°C. The development of ferrous sulfide (black ppt.) confirmed the positive test results. Oxidase tests were carried out as described by Hayward (1960). In this test, 24 h old bacterial culture was mixed with a few drops of 1% N,N,N′,N′-tetramethyl-p-phenylenediamine (TMPD) solution (Sigma-Aldrich, United States) on Whatman No. 1 filter paper. The appearance of a dark purple color within 30 s confirmed the positive test results. The test for oxidative fermentation was performed as described by Hugh and Leifson (1953), while nitrate reduction and gelatin hydrolysis assays were carried out using the protocol previously used by Thankamani and Dev (2011).

### Molecular Characterization of Rhizobacterial Isolates

Bacterial agents displaying promising antagonistic activity were identified using 16S rRNA gene sequencing (Kumar et al., 2015). Total genomic DNA was extracted from bacterial isolates, by using the GeneJet Genomic DNA purification Kit (Thermo Scientific Waltham, United States) following the manufacturer’s instructions. The 16S rRNA region was amplified in a polymerase chain reaction (PCR) using primer pair 27F [5′ -AGAGTTTGATC-MTGGCTCAG- 3′] and 1492R [5′ -GGTTACCTTGTTAC-GACTT- 3′], respectively (Habib et al., 2016), in 50 μl reactions consisting 25–150 ng of DNA template, 1X of Taq buffer (10 mM Tris pH 9, 50 mM KCl, 0.01% gelatin), 200 μM of each dNTP, 1.25 mM of MgCl_2_, 0.4 μM of each primer, and 0.5 U of Taq DNA polymerase (Qiagen, Germany).

Polymerase chain reaction conditions were: initial denaturation of DNA template at 95°C for 1 min per cycle, 35 cycles of denaturation at 95°C for 15 s, annealing at 55°C for 15 s, extension at 72°C for 1 min and a final elongation at 72°C for 7 min. Amplified DNA products were then run on 1% (w/v) agarose gel and visualized under UV transilluminator after staining with Ethidium bromide (EB). PCR products (1.5 Kb) of 16S rRNA gene were cleaned with Gel and PCR Clean-Up System (Promega, United States), and quantified by NenoDrop.

The amplified DNA products were then sent for sequencing to the Department of Crop Sciences, University of Illinois, Urbana, IL, United States. Frequents were sequenced using 27F and 1492R primers, and obtained sequences were joined by Bioinformatics software for life science (DNASTAR software). Sequences were run in the BLAST program^1^ at the National Center for Biotechnology Information (NCBI) server to search the closely related sequences. All the retrieved sequences along with tested bacterial isolates sequences were aligned together using CLUSTAL W Program.

The evolutionary relatedness between the tested bacterial sequences and retrieved sequences was determined by constructing a phylogenetic tree using the neighbor-Joining (N-J) method in Molecular Evolutionary Genetics Analysis software MEGA X version 10.1.7 with 1000 bootstrap replicates. The evolutionary distances were calculated using the Kumara 2-parameter model (K2 + G) (Kumar et al., 2018). The 16S rRNA gene sequences were deposited in the GenBank nucleotide database and accession numbers were obtained.

## Characterization of Isolated Bacterial Strain for Plant Growth Promoting Traits

### Ammonia (NH_3_) Production

The production of NH_3_ was tested in accordance with Cappuccino and Sherman (2005). In particular, 24 h old each bacterial isolate (100 μl), grown on nutrient broth medium was inoculated on test tubes containing peptone water (10.0 g peptone; 5.0 g NaCl; 1000 mL distilled water; 7.0 pH) and incubated at 28°C for 48–72 h. 500 μl of Nessler’s reagent (Fisher^®^, United States) was added to each test tube. Brown to yellow color development confirmed the NH_3_ production.

### Starch Hydrolysis

A starch hydrolysis test was performed using the protocol previously described (Marten et al., 2000). In particular, 24 h old bacteria were cultured on LB agar medium containing Petri plates amended with 2% starch and incubated at 30 ± 2°C for 48–72 h. Plates were then flooded with Lugol’s solution. Clear halo zone formation around the bacterial growth confirmed the positive test results of starch hydrolysis.

### Phosphate Solubilization

Phosphate solubilizing ability was assessed by following the procedure previously reported by Verma et al. (2001). For this test, the bacterial cultures were streaked on Pikovskaya’s agar medium (HiMedia Laboratories, India) and supplied with tricalcium phosphate in Petri plates. Plates were then incubated at 28 ± 2°C for 72–96 h. The formation of clear halo zones encircling the bacterial colonies indicated phosphate solubilization. Phosphate solubilization was quantified by Phosphomolybdate blue color assay as previously described by Murphy and Riley (1962).

### Hydrogen Cyanide

Production of HCN was assessed by adopting the procedure reported by Lorck (1948). In this test, 24 h old bacterial strains were streaked on NA medium containing Petri plates amended with glycine (4.4 gL^–1^). Agar medium was covered with Whatman number 1 filter paper previously dipped in a solution of 0.5% picric acid and 2% sodium carbonate (w/v). Petri plates were Parafilmed, and incubated at 28 ± 2°C for 96 h. The development of an orange or red color indicated HCN production.

### Indole Acetic Acid (IAA) Detection and Quantification

Bacterial isolates were inoculated on LB medium amended with 0.5 mgL^–1^ tryptophan/mL, incubated at 28 ± 2°C for 5 days, and centrifuged at 3,000 rpm for 30 min. The supernatant (2 mL of the aliquot) was added with two drops of orthophosphoric acid and 4 mL of Salkowaski’s reagent (150 mL concentrated H_2_SO_4_, 250 mL distilled water, 7.5 mL 0.5 M FeCl_3_.6H_2_O), and incubated at room temperature in dark for 20 min (Gordon and Weber, 1951). The development of a pink-red color indicated the production of IAA. The absorbance of IAA was recorded at 530 nm using a spectrophotometer (Thermo Scientific, United States) and the concentration of IAA was measured against a standard curve developed from pure IAA solution. There were three replications for each bacterial isolate, and all the mean values were statistically analyzed.

### Siderophores Production

Siderophores production was tested in accordance with Schwyn and Neilands (1987). In particular, the siderophores production test was performed by culturing bacterial isolates (10^8^cfu mL^–1^) on Chrome azurol S agar medium followed by incubation at 28 ± 2°C for 72 h. Change in the color from yellow to orange was an indication of siderophores production. Siderophores production was quantified by CAS-liquid assay (Payne, 1994); Optical density (OD) was measured at 630 nm against a reference consisting of CAS reagent. Siderophore contents were calculated by the formula:

%siderophoreunit=Ar-AsArX 100

Ar = absorbance of the standard at 630 nm.

As = absorbance of the sample at 630 nm.

### Multiple Antibiotic Resistance of Rhizobacterial Isolates

Multiple antibiotic resistance tests were performed to check the level of susceptibility and resistance of rhizobacterial isolates by following the methodology previously described by Singh et al. (2013). The test was performed to screen the bacterial isolates against streptomyces, ampicillin, rifampicin, penicillin G, and vancomycin at different concentration levels (0 ppm, 100 ppm, 200 ppm, 300 ppm, 400 ppm, and 500 ppm) *in vitro*. For this, 100 μl of 24 h old bacterial suspensions prepared in NB medium were spread on Petri plates containing solid NA medium. Small filter paper disks immersed in each antibiotic concentration were placed on the media and plates were incubated at 26 ± 2°C for 24 h. Each treatment was replicated five times and the zone of inhibition was measured from each treatment.

## In-Planta Assays

### Effect of Seed Bacterization on Germination and Vigor Index in Chili

For this, chili seeds (variety: Long green) were bacterized by immersing surface sterilized seeds in 24 h old bacterial inoculum prepared in 25 mL LB medium (bacterial concentrations 10^6^, 10^7^, and 10^8^ cfu mL^–1^) by gently shaking on a shaker for 2 h. Ten seeds/Petri plates were placed on two layers of moistened filter paper in each 9-cm plate and were incubated at 28 ± 2°C in the growth room (Seleim et al., 2011). Filter papers were kept moist by adding 5 mL autoclaved distilled water when needed. Seeds soaked in only autoclaved distilled water were kept as control. Data on growth parameters and vigor index was recorded after 20 days of incubation. The experiment was performed with five replications for each treatment. Seed germination percentage (GP) and vigor index (VI) were recorded by the formulas:

Seedgermination(%)=No.of⁢germinated⁢seedstotal⁢no.of⁢seedsX 100

Vigorindex=%seedgerminationXtotalplantlength

### Evaluation of Bacterial Isolates for the Induction of Defense Related Enzymes

Bacterial antagonists were evaluated for defense related enzyme induction ability in a pot experiment on chili (variety: Long green) under natural conditions in a net house. Plastic pots of 1.5 L capacity were filled with autoclaved sandy loam textured soil and flooded with 20 mL sporangial suspension of *P. myriotylum* (1x10^3^ sporangia/mL). Fifteen days old healthy seedlings were dipped for 2 h in overnight bacterial suspension (10^8^ cfu/mL) in LB medium before shifting in pots containing infested soil. Five seedlings per pot were sown in three repeats and placed under net house conditions at 28 ± 2°C and 80% relative humidity. The experiment was performed with ten treatments viz., T1 (*P. myriotylum* as negative control), T2 (4a2 – *Flavobacterium* spp.), T3 (JHL-8 – *Bacillus megaterium*), T4 (JHL-12 – *P. putida*) T5 (1C2 – *B. cereus*), T6 (RH-24 – *B. subtilis*), T7 (1D – *B. cereus*), T8 (5C – *P. putida*) T9 (RH-87 – *P. libanensis*) T10 (Untreated control) in three repeats. Root tissues were taken at 1, 3 and, 5 days intervals after transplant.

### Enzyme Extraction and Quantification

Representative chili root samples (2 g) were crushed with 4 mL of 0.1 M sodium phosphate buffer at 4°C in sterilized mortar and pestle. The homogenized solution was centrifuged for 15 min at 10,000 rpm and 4°C and supernatant was used for the estimation of PO, PPO, PAL, and chitinase activity by spectrophotometry (Anand et al., 2007).

### Peroxidase Test (PO)

Peroxidase test activity was tested by following the methodology adopted by Hammerschmidt et al. (1982). In this test, peroxidase activity was measured by mixing 0.5 mL of enzyme extract with 1.5 mL of pyrogallol (0.05 M) and 0.5 mL of 1% H_2_O_2_ and incubated at room temperature. The absorbance change was noted at 420 nm at 30 s intervals for 3 min against a blank.

### Polyphenol Oxidase Test

Polyphenol oxidase test was performed in accordance with the methodology described by Mayer et al. (1966). In particular, PPO activity was determined by mixing 200 mL of the crude enzyme extract with 1.5 mL of 0.1 M sodium phosphate buffer, 200 mL of 0.01 M catechol was added to start the reaction, and absorbance was recorded at 495 nm wavelength.

### Phenylalanine Ammonialyas Test (PAL)

A PAL activity test was performed in accordance with Whetten and Sederoff (1992). In particular, PAL activity was assessed by mixing 100 μl of enzyme, 500 μl of 50 mM Tris HCL, and 600 μl of 1 mM L-phenylalanine followed by incubation for 1 h. The reaction was stopped by adding with 2N HCL followed by adding 1.5 mL toluene in the mixture, vortexed for 30 s, and centrifugation at 1000 rpm for 5 min. Toluene fraction carrying trans-cinnamic acid was separated. The toluene phase was estimated at 290 nm wavelength against the toluene as blank, and a standard curve was constructed with graded amounts of cinnamic acid in toluene.

### Testing of Bacteria for Disease Suppression and Plant Growth Promotion (PGP) Traits in Pot Trials

The potential of antagonistic bacteria to suppress the damping-off disease and plant growth promotion effect was tested under natural environmental conditions in net house conditions in a repeated experiment. In this study, zoospores of *P. myriotylum* were obtained by following the procedure previously described by Rahimian and Banihashemi (1979), and concentration was maintained at 1 x 10^6^ zoospores/mL using a hemocytometer. Plastic pots (1.5 L) were filled with sterilized soil/peat (75%: 25% ratio) and 100 mL zoospore suspension of *P. myriotylum* was added in the soil before sowing the chili seeds (Variety: Long green). Prior to sowing, surface sterilized chili seeds were soaked individually for 2 h in bacterial suspensions (10^8^ cfu/mL) prepared in Luria-Bertani (LB) medium (Sigma-Aldrich, United States). Un-inoculated seeds without *P. myriotylum* and rhizobacteria were kept as untreated control (UTC) while the seeds inoculated with *P. myriotylum* were kept as a negative control (NC). All other plant management practices were kept the same for all the treatments. Experiments were carried out with five replications for each treatment. Pots were kept under net house conditions and damping-off disease incidence and seed germination percentage were recorded after 15 days of sowing while data on PGP was taken 30 days after sowing the seeds.

### Statistical Data Analysis

Statistical data analysis was performed using Statistix 8.1 software and MS Excel 2010. All the experiments were performed in a completely randomized design (CRD) with replicated treatments. All the experiments were repeated at least two times to confirm the results. Mean values for each treatment were calculated, and all the treatment means were compared via the Analysis of variance (ANOVA) test using the least significant differences (LSD) at 5% probability (*P* ≤ 0.05). The correlation was studied in Microsoft Office Excel 2010.

## Results

### Pathogen Inoculum of *P. myriotylum*

**FIGURE 1:**
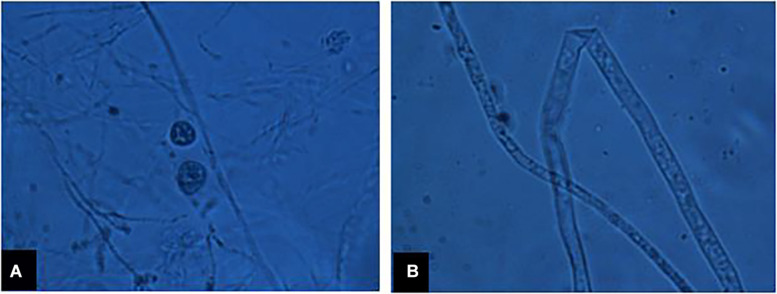
**(A)** Sexual fruiting bodies of *P. myriotylum*, **(B)** coenocytic fungal hyphae under microscope.

A total of 13 isolates of *P. myriotylum* were recovered from infected chili roots showing characteristic symptoms of damping-off disease on corn meal agar medium (CMA). They were identified based on morphological characters, i.e., coenocytic hyphae bearing lobate sporangia (7 to 15 μm wide), knob-like appressorium, vesicles (43 to 52 μm in diameter) bearing 29 to 45 zoospores/vesicle, encysted zoospores (10 to 12 μm in diameter), terminal oogonia (30 to 38 μm in diameter), crooked necked antheridia (4 to 7 antheridia per oogonium), and aplerotic oospores (25 to 31 μm in diameter) as presented in Figure 1. Internal transcribed spacer regions (ITS1 and ITS2) were amplified by PCR and final sequences were submitted to the GenBank database under the accessions MF143429 and MF143430. BLAST analysis of approximately 700 bp fragments showed 99% sequence identity with an already published sequence of *P. myriotylum* (accession HQ643704). Both the isolates PMyr-1 and PMyr-2 produced characteristic damping-off symptoms in pathogenicity tests on chili. This pathogen was previously described in the first report on *P. myriotylum* causing damping-off and root rot in chili from Punjab, Pakistan.

### Isolation and *in vitro* Screening of Bacterial Isolates Against *P. myriotylum*

**FIGURE 2:**
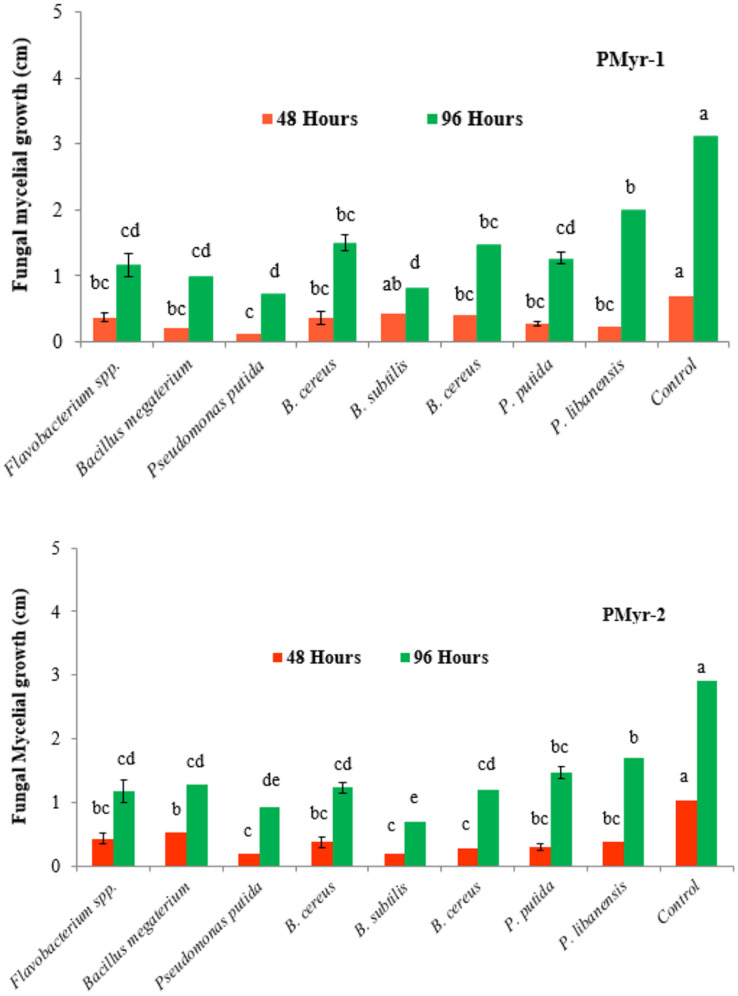
*In vitro* antagonistic activity of rhizobacteria isolated from chili growing fields against *P. myriotylum*, the causal agent of damping-off in chili. Fungal mycelial growth was measured in cm from each treatment. PMyr-1 and PMyr-2 are two virulent strains of *P. myriotylum*. Presented values are the average of three replicates of each treatment. Error bars show standard deviations and letters on each bar represent the significant difference among the values at 5% level of significance.

A total of 110 rhizobacterial isolates were recovered from the healthy chili roots and rhizospheric soil samples and were screened for antagonistic potential against two highly virulent strains of *P. myriotylum* (PMyr-1 and PMyr-2) isolates in repeated dual culture experiments on the PDA medium. Out of all tested isolates, 28 (25.5%) bacterial isolates exhibited varied levels of antagonistic activities (Unpublished data). Data recorded after 48 and 96 h of incubation showed that out of 28 bacterial isolates, 8 (28.6%) rhizobacteria; *Flavobacterium* spp., *Bacillus megaterium*, *Pseudomonas putida*, *B. cereus*, *B. subtilis*, *B. cereus*, *P. putida*, and *P. libanensis* exhibited significant antagonistic activity against *P. myriotylum* as compared to control under *in vitro* conditions (Figure 2). Forty-eight hours after inoculation, mycelial growth inhibition percentage in PMyr-1 was ranged from 38.6 to 81.4% (48 h after inoculation) and 36.1 to 76.7% (96 h after inoculation), while percentage mycelial growth inhibition in PMyr-2 was 48.5 to 80.6% and 41.4 to 75.9% after 48 and 96 h of inoculation, respectively. These eight potential bacterial isolates were further tested by subsequent *in vitro* experiments.

### Biochemical Featuring of Rhizobacterial Isolates

The response of all the tested bacterial isolates toward various biochemical tests is presented in Table 1. Out of the eight bacterial isolates, four isolates *B. megaterium*, *B. cereus*, *B. subtilis*, and *B. cereus* were gram positive while three isolates gave fluorescence emission. Four bacteria including *Flavobacterium* spp., *Pseudomonas putida*, *P. putida*, and *P. libanensis* gave positive results for the KOH solubility test while all the bacteria isolates were positive for catalase production. In response to the levan production test, two bacterial isolates, *B. cereus* and *P. libanensis* showed positive test results while *P. putida* and *B. cereus* were positive for carbohydrate fermentation reaction. All the tested bacterial isolates showed positive results for the oxidase test except *P. libanensis*, which was not tested for the response. All the bacterial antagonists except *B. subtilis* exhibited a positive response in the oxidative fermentative test. All bacterial isolates except for *Flavobacterium* spp. gave positive results for the nitrate reduction and gelatin hydrolysis tests. Nitrate reduction and gelatin hydrolysis tests were not performed on *P. libanensis* and *Flavobacterium* spp. bacterial isolates, respectively.

**TABLE 1:** Response of rhizobacteria isolated from chili growing fields to various biochemical tests.

**Bacterial isolates**	**GS**	**FE**	**KOH**	**CAT**	**LP**	**CF**	**OXD**	**OFR**	**NR**	**GH**
*Flavobacterium* spp.	−	−	+	+	−	−	+	+	−	NA
*B. megaterium*	+	−	−	+	−	−	+	+	+	+
*P. putida*	−	+	+	+	−	+	+	+	+	+
*B. cereus*	+	−	−	+	−	+	+	+	+	+
*B. subtilis*	+	−	−	+	−	−	+	−	+	+
*B. cereus*	+	−	−	+	+	+	+	+	+	+
*P. putida*	−	+	+	+	−	−	+	+	+	+
*P. libanensis*	−	+	+	+	+	−	NA	+	NA	+

*Each test result was confirmed in three replications for each bacterial isolate. (+) positive reaction (−) negative reaction and (NA) not tested results for: gram staining (GS), fluorescence emission (FE), potassium hydroxide (KOH), catalase test (CAT), levan production (LP), carbohydrate fermentation (CF), oxidase test (OXD), oxidative fermentative (OFR), nitrate reduction (NR), gelatin hydrolysis (GH) test.

### Molecular Characterization of Rhizobacterial Isolates

**FIGURE 3:**
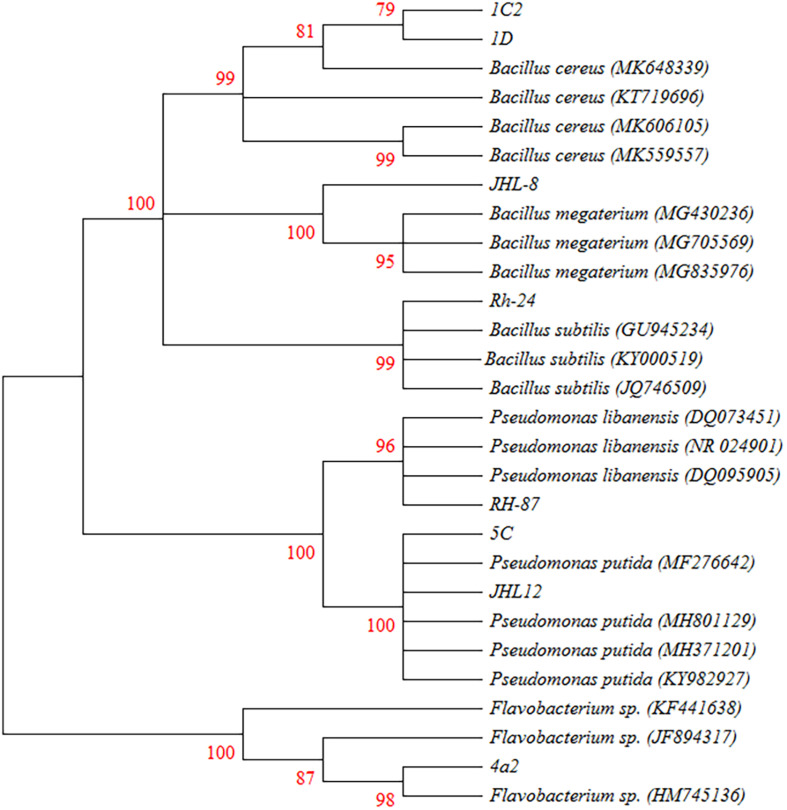
Phylogenetic tree based on the 16S rRNA sequences (≈1500 bp) showing the relationships between the representative rhizobacteria and closely associated neighbors. 16s rRNA gene fragments were amplified by PCR protocols using 27F and 1492R primers and amplified products were confirmed by Gel electrophoresis against 1 kb ladder. Gel purified DNA products were sent for sequencing and obtained sequences were joined by using DNASTAR software. BLAST analysis was performed to retrieve closely associated bacterial sequences. All the Sequences were aligned in CLUSTAL W program and the phylogenetic tree was constructed using Kumara 2-parameter model (K2 + G) using 1000 replicates as bootstrap values and >70% are labeled.

Phylogenetic analysis from 16S rRNA sequences (≈ 1500 bp) of eight rhizobacterial isolates showed that all the tested bacteria belonged to *Bacillus*, *Pseudomonas*, and *Flavobacterium* spp. (Figure 3). Bacterial isolates ID and 1C2 had 97 to 98% sequence homology with *Bacillus cereus* (accessions MK606105 and MK648339, respectively) and the sequences of ID and 1C2 were submitted to the GenBank database under accession numbers MH393211 and MH393210.1, respectively. The 16S rRNA gene sequence of the bacterial isolate JHL-8 (accession MH393209) showed 99% sequence similarity with *B. megaterium* (accession MG430236). The sequence of RH-24 (accession MH393208) had 99% sequence homology with *B. subtilis* (accession KY000519). Isolate RH-87 (accession MT421780) was closely related to *Pseudomonas libanensis* 99% identity with GenBank accession number DQ095905. Bacterial isolates 5C and JHL-12 had 99% sequence homology with *P. putida* (accessions KY982927 and MF276642, respectively) and the sequences of 5C and JHL-12 were deposited to the GenBank database under accession numbers MH371201 and MH371200, respectively. Bacterial isolate 4a2 (accession MT421823) displayed 99% identity with the GenBank sequence of *Flavobacterium* spp. (accession HM745136). The accession numbers of all the tested bacterial antagonists and sequence homology percentage with their reference isolates are given in Table 2.

**TABLE 2:** Sequence analysis of 16S rRNA from rhizobacteria isolated from chili growing fields and their homology with the reference bacteria.

**Isolate ID**	**Sequence (bp)**	**Accession No.**	**Identified As**	**Identity with Accession No.**	**Similarity Index**
*Flavobacterium* spp.	1377	MT421823	*Flavobacterium* spp.	HM745136	99.7%
*B. megaterium*	948	MH393209	*Bacillus megaterium*	MG430236	99%
*P. putida*	1316	MH371200	*Pseudomonas putida*	MF276642	99%
*B. cereus*	938	MH393210	*B. cereus*	MK648339	98%
*B. subtilis*	1381	MH393208	*B. subtilis*	KY000519	99%
*B. cereus*	1088	MH393211	*B. cereus*	MK606105	97%
*P. putida*	1417	MH371201	*P. putida*	KY982927	99%
*P. libanensis*	1370	MT421780	*P. libanensis*	DQ095905	99%

### Characterization of Rhizobacterial Isolates for Biocontrol and Plant Growth Promotion (PGP) Traits

All the rhizobacterial isolates were tested for biocontrol and plant growth promotion (PGP) traits and their responses are given in Table 3. All the bacterial isolates displayed positive test results for ammonia production as indicated by the brown to yellow color development. All the bacterial isolates were able to hydrolase starch while the test was not performed for *Flavobacterium* spp. All the tested bacterial isolates except *B. cereus* exhibited positive test results for hydrogen cyanide (HCN) production, and the said test was not performed for *Flavobacterium* spp. A halo zone formation around the bacterial growth on Pikovskaya’s agar medium after 96 h indicated the positive test results for phosphate solubilization by the bacterial isolates. *Bacillus* spp. *Bacillus megaterium* showed the highest P-solubilization (103 μgmL^–1^) followed by *B. subtilis* (97 μgmL^–1^). In the case of *Pseudomonas* sp., *P. putida* exhibited maximum P-solubilization (84 μgmL^–1^) followed by *P. libanensis* (79 μgmL^–1^) and *P. putida* (75 μgmL^–1^). *Flavobacterium* spp. showed maximum P-solubilization (86 μgmL^–1^) and the test was not done for bacterial isolate *B. cereus*. All the bacterial isolates showed pink-red color development as an indication of IAA production. The spectrophotometry study confirmed the production of IAA by bacterial isolates between 39 and 13.4 μgmL^–1^. Maximum IAA (39 μgmL^–1^) was produced by *B. subtilis* followed by *Flavobacterium* spp. (34.1 μg mL^–1^), *B. megaterium* and *P. putida* (26.7 μgmL^–1^) while the minimum amount of IAA (13.4 μgmL^–1^) was produced by *P. libanensis*. Siderophore production was indicated by the change in color from blue to orange on chrome azurol S agar medium. *Bacillus. subtilis* showed the highest siderophore production (27.7%) followed by *B. megaterium* (23.7%) and *B. cereus* (23.3%). *P. libanensis* showed the highest siderophores production (23.6%) and *P. putida* (19%) while *Flavobacterium* spp. showed 18.5% siderophores production.

**TABLE 3:** Characterization of rhizobacteria isolated from chili growing fields for plant growth promoting (PGP) traits.

**Bacterial isolates**	**AP**	**SH**	**HCN**	**P-Solubilization (μg mL^–1^)**	**IAA production (μg mL^–1^)**	**SDP (%)**
*Flavobacterium* spp.	+	ND	ND	86 ± 4.33^b^	34.1 ± 1.7^a^	18.5 ± 1.1^d^
*B. megaterium*	+	+	+	103 ± 3.53^a^	26.7 ± 2.3^b^	23.7 ± 2.0^b^
*P. putida*	+	+	+	75 ± 2.89^c^	19.6 ± 1.4^c^	17.3 ± 1.4^d^
*B. cereus*	+	+	+	81 ± 3.28^bc^	13.9 ± 1.7^cd^	23.3 ± 0.8^bc^
*B. subtilis*	+	+	+	97 ± 4.36^a^	39.0 ± 1.5^a^	27.7 ± 1.5^a^
*B. cereus*	+	+	−	ND	19.3 ± 2.8^cd^	17.1 ± 0.9^d^
*P. putida*	+	+	+	84 ± 2.31^bc^	26.7 ± 2.2^b^	19.0 ± 1.7^cd^
*P. libanensis*	+	+	+	79 ± 3.93^bc^	13.4 ± 1.5^d^	23.6 ± 1.6^ab^
LSD				10.062	5.8787	4.2705

*(+) positive (−) negative test, AP (ammonia production), SH (starch hydrolase), HCN (hydrogen cyanide production), PHS (phosphate solubilization), IAA (indole acetic Acid production), SDP (siderophore production %). Data are average values of three replicates for each bacterial isolate. (±) standard error values.*

### Multiple Antibiotic Resistance of Rhizobacterial Isolates

**FIGURE 4:**
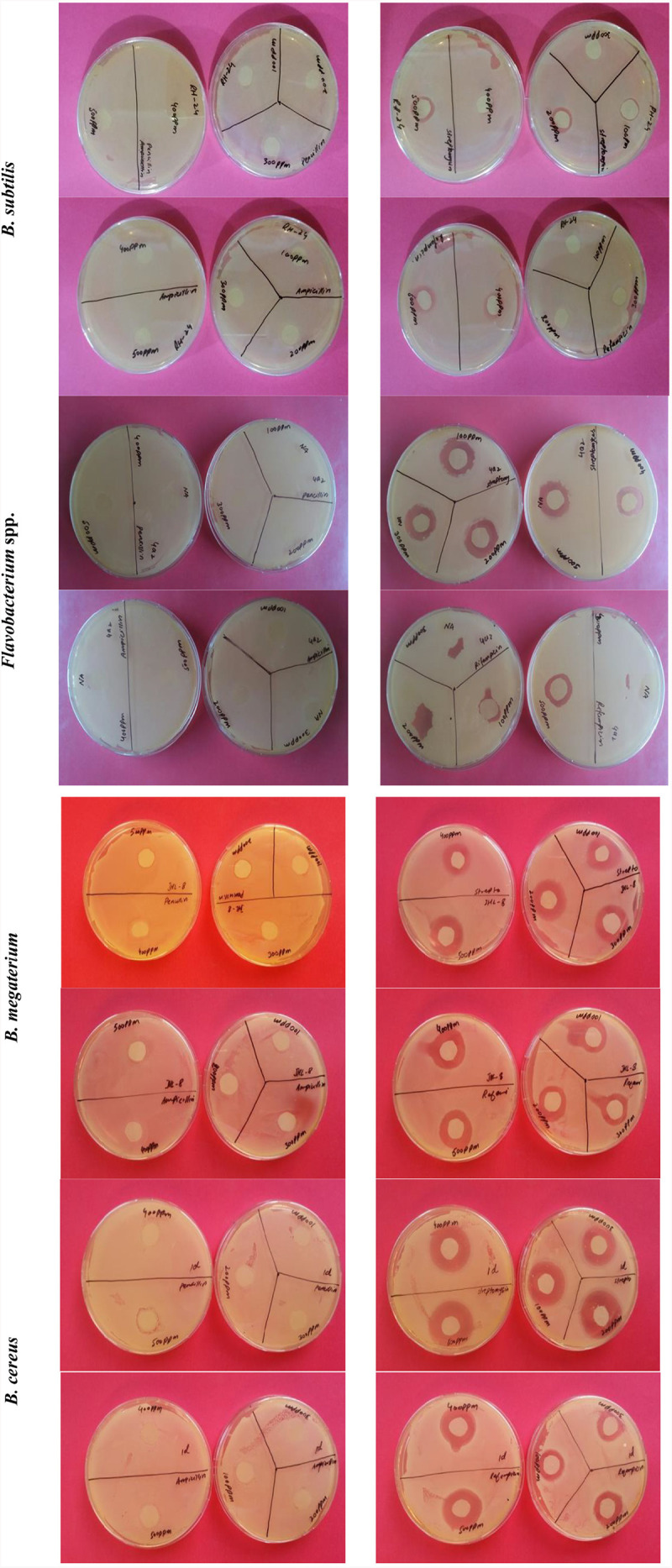
Multiple antibiotic resistance assays to assess the resistance and susceptibility levels of rhizobacteria against Streptomyces, Ampicillin, Rifampicin, Penicillin G, and Vancomycin at 0, 100, 200, 300, 400, and 500 ppm concentrations. Zone of inhibition (cm) was measured from each replicated plate 24 h after incubation.

**FIGURE 5:**
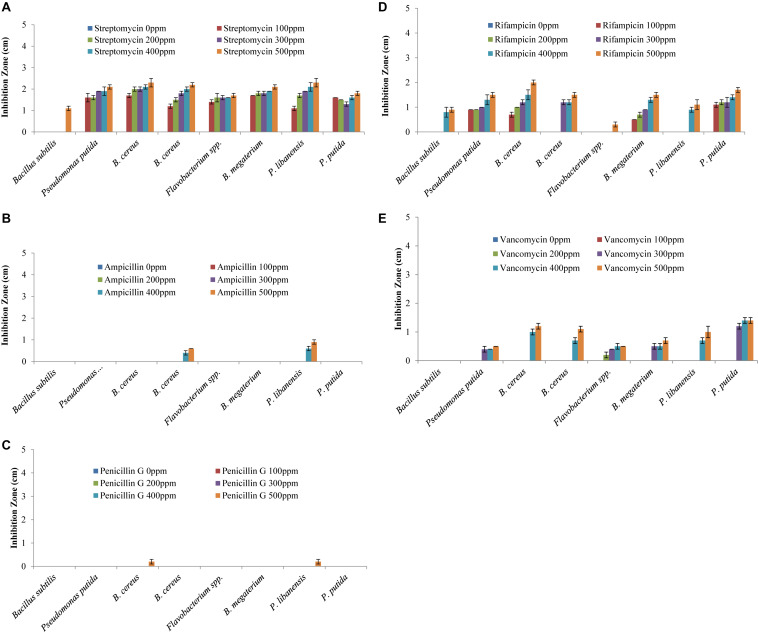
Multiple antibiotic resistance assays to test the resistance and susceptibility levels of rhizobacteria against Streptomyces **(A)**, Ampicillin **(B)**, Rifampicin **(C)**, Penicillin G **(D)**, and Vancomycin **(E)** at six different concentration levels. Each treatment was tested in five replications and bar graphs are made by using average values for each treatment. Error bars on each bar represent the standard error (SE).

All the bacteria displayed varying levels of resistance and susceptibility against all tested antibiotics (Figure 4) and zone of inhibition around the bacterial cultures were measured (Figures 5A–E). All the rhizobacterial isolates showed no tolerance against Streptomyces at all dose levels except *Bacillus subtilis*, which showed resistance up to 400 ppm dose level. Against Ampicillin, all the tested bacteria showed resistance to all the dose levels except *B. cereus* and *P. libanensis* which showed little susceptibility at the two highest dose levels. When tested against Penicillin G, bacterial isolates, *B. subtilis*, *P. putida*, *B. cereus*, *Flavobacterium* spp., *B. megaterium*, and *P. putida* were found to have resistance at all the dose levels while *B. cereus* and *P. libanensis* showed little susceptibility at a 500 ppm dose level. Most of the tested bacteria showed no resistance against Rifampicin at all six dose levels, however, *B. subtilis* showed resistance against Rifampicin up to 300 ppm dose level and *Flavobacterium* spp. showed little susceptibility against Rifampicin at 500 ppm. *Bacillus subtilis* showed the highest tolerance against Vancomycin at all the dose levels while all other tested bacterial isolates showed varying levels of susceptibility response at different dose levels. The tested isolates ID, *B. cereus* and *P. libanensis* showed resistance response up to 300 ppm dose level of Vancomycin while the bacterial isolates *Flavobacterium* spp., *B. megaterium*, and *P. putida* showed maximum susceptibility.

## In-Planta Assays

### Effect of Bacterial Inoculants on Chili Seed Germination

**FIGURE 6:**
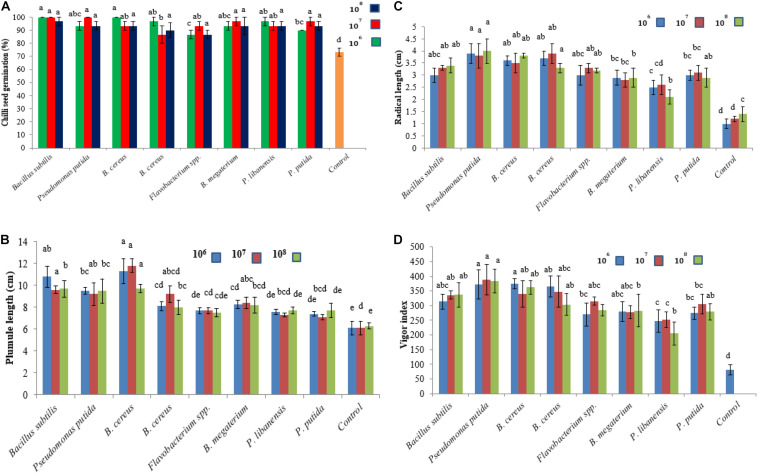
Effect of rhizobacterial inoculants on chili seeds **(A)** seed germination percentage, **(B)** plumule length, **(C)** radical length, **(D)** vigor index. Chili seeds were treated with bacterial strains belonging to *Flavobacterium* spp. *Pseudomonas* spp. and *Bacillus* spp. while the seeds in the control treatment were dipped in double sterilized water only. Seeds were grown on moist Whatman filter paper No. 41 in sterile Petri plates under controlled conditions. Each treatment was replicated three times and bars are made from the average of three values. Error bars on each bar represent the standard error (SE).

The effect of bacterial seed treatment upon seed germination and plant growth parameters (PGP) varied with different bacterial isolates (Figures 6A–D). All the bacterial isolates produced significant effects on seed germination percentage and PGP compared to control treatment. None of the tested bacterial isolate at any applied concentration level showed a reduction in seedling germination and phytotoxic effects on chili seedlings. Maximum plumule length was recorded 11.8 cm in chili seedling inoculated with *B. cereus* at 10^7^ cfu followed by *B. subtilis* (9.6 cm) over control. The maximum radical length was recorded 4 cm for *P. putida* at 10^8^ cfu followed by *B. cereus* while the minimum radical length was recorded for *P. libanensis* at all the tested concentrations compared to control. Vigor index was significantly increased in bacterized chili seed over untreated control.

### Induction of Defense Related Enzymes in Chili Plants

All the tested bacterial isolates significantly induced defense related enzymes in chili seedlings under the pathogen presence (Table 4). In chili seedlings treated with rhizobacterial suspensions, an increase in Peroxidase (PO) activity was observed 3 and 5 days after inoculation (DAI). Maximum PO activities were recorded in seedlings treated with *Flavobacterium* spp. followed by *B. megaterium* and *B. subtilis* and PO activates were recorded almost three folds higher than the negative control (NC) and untreated control (UC). The increased activity of Polyphenol oxidase (PPO) was observed on the fifth day after inoculation in seedlings treated with *B. subtilis* followed by *Flavobacterium* spp. and *P. putida* as compared to negative and untreated control. Phenylalanine ammonia-lyase (PAL) activities were observed high in all the chili seedlings treated with bacterial isolates. Maximum PAL activates were observed in seedlings inoculated with *B. subtilis* followed by *B. megaterium* and *B. cereus*. Phenylalanine ammonia-lyase (PAL) activities were recorded almost three-fold higher in all the bacterial treated seedlings compared to negative and untreated controls.

**TABLE 4:** Induction of defense related enzymes in chili seedlings inoculated with antagonistic rhizobacterial isolates.

**Trt.**	**PO activity (Katal/mg of total proteins)**			**PPO activity (Katal/mg of total proteins)**			**PAL activity (Katal/mg of total proteins)**		
	**1 DAI**	**3 DAI**	**5 DAI**	**1 DAI**	**3 DAI**	**5 DAI**	**1 DAI**	**3 DAI**	**5 DAI**
NC	0.23 ± 0.03^c^	0.37 ± 0.03^c^	0.33 ± 0.03^c^	0.13 ± 0.03^f^	0.30 ± 0.06^d^	0.40 ± 0.06^e^	0.23 ± 0.03^a^	0.37 ± 0.07^d^	0.40 ± 0.06^e^
*Flavobacterium* spp.	0.37 ± 0.03^ab^	1.06 ± 0.05^a^	1.19 ± 0.05^a^	0.43 ± 0.03^bc^	1.50 ± 0.06^b^	1.77 ± 0.19^b^	0.36 ± 0.02^ab^	1.10 ± 0.21^bc^	1.38 ± 0.10^cd^
*B. megaterium*	0.33 ± 0.03^bc^	0.93 ± 0.03^a^	1.17 ± 0.03^a^	0.45 ± 0.08^ab^	1.33 ± 0.09^b^	1.30 ± 0.06^c^	0.44 ± 0.07^a^	1.33 ± 0.30^abc^	1.67 ± 0.14^abc^
*P. putida*	0.33 ± 0.03^bc^	0.70 ± 0.06^b^	0.87 ± 0.03^b^	0.23 ± 0.03^def^	1.53 ± 0.12^b^	1.57 ± 0.15^bc^	0.43 ± 0.15^a^	1.42 ± 0.10^ab^	1.46 ± 0.10^bc^
*B. cereus*	0.30 ± 0.06^bc^	0.67 ± 0.03^b^	0.86 ± 0.02^b^	0.20 ± 0.06^ef^	1.57 ± 0.22^b^	1.61 ± 0.13^bc^	0.45 ± 0.01^a^	1.53 ± 0.23^ab^	1.75 ± 0.14^ab^
*B. subtilis*	0.47 ± 0.03^a^	1.03 ± 0.03^a^	1.17 ± 0.03^a^	0.60 ± 0.06^a^	2.03 ± 0.15^a^	2.43 ± 0.24^a^	0.53 ± 0.09^a^	1.70 ± 0.21^a^	1.90 ± 0.21^a^
*B. cereus*	0.27 ± 0.03^bc^	0.63 ± 0.07^b^	0.78 ± 0.04^b^	0.33 ± 0.09^bcde^	1.47 ± 0.09^b^	1.52 ± 0.07^bc^	0.40 ± 0.04^ab^	1.53 ± 0.07^ab^	1.58 ± 0.02^abc^
*P. putida*	0.27 ± 0.03^bc^	0.67 ± 0.07^b^	0.81 ± 0.01^b^	0.37 ± 0.03^bcd^	1.67 ± 0.15^ab^	1.77 ± 0.05^b^	0.46 ± 0.03^a^	1.23 ± 0.07^abc^	1.40 ± 0.03^bcd^
*P. libanensis*	0.27 ± 0.07^bc^	0.77 ± 0,03^b^	0.88 ± 0.04^b^	0.22 ± 0.02^*def*^	1.40 ± 0.17^b^	1.33 ± 0.09^c^	0.38 ± 0.04^ab^	1.26 ± 0.25^abc^	1.56 ± 0.19^abc^
UC	0.29 ± 0.07^bc^	0.41 ± 0.06^c^	0.42 ± 0.04^c^	0.29 ± 0.05^*cde*^	0.69 ± 0.08^c^	0.79 ± 0.05^d^	0.43 ± 0.03^a^	0.87 ± 0.06^cd^	1.06 ± 0.12^d^
LSD	0.132	0.145	0.105	0.155	0.376	0.368	0.189	0.524	0.367

*Defense related enzymes viz., peroxidase (PO), polyphenol oxidase (PPO), and phenylalanine ammonia-lyase (PAL) were studied. Sterilized soil was flooded with sporangial suspension of *P. myriotylum* before transplanting the chili seedlings. Fifteen days old chili seedlings were inoculated with rhizobacterial suspension (10^8^ cfu/mL) for 2 h before transplanting in sick soil containing pots. Root samples were taken 1, 3, and 5 days after transplant. Only *P. myriotylum* treated pots were considered as negative control (NC) while non-inoculated plants with pathogen were kept us untreated control (UC). Means are an average of three repeats and (±) indicate the standard error (SE). DAI: days after inoculation.*

### Testing of Rhizobacterial Isolates for Disease Suppression and Plant Growth Promotion Traits in Pot Trials

**FIGURE 7:**
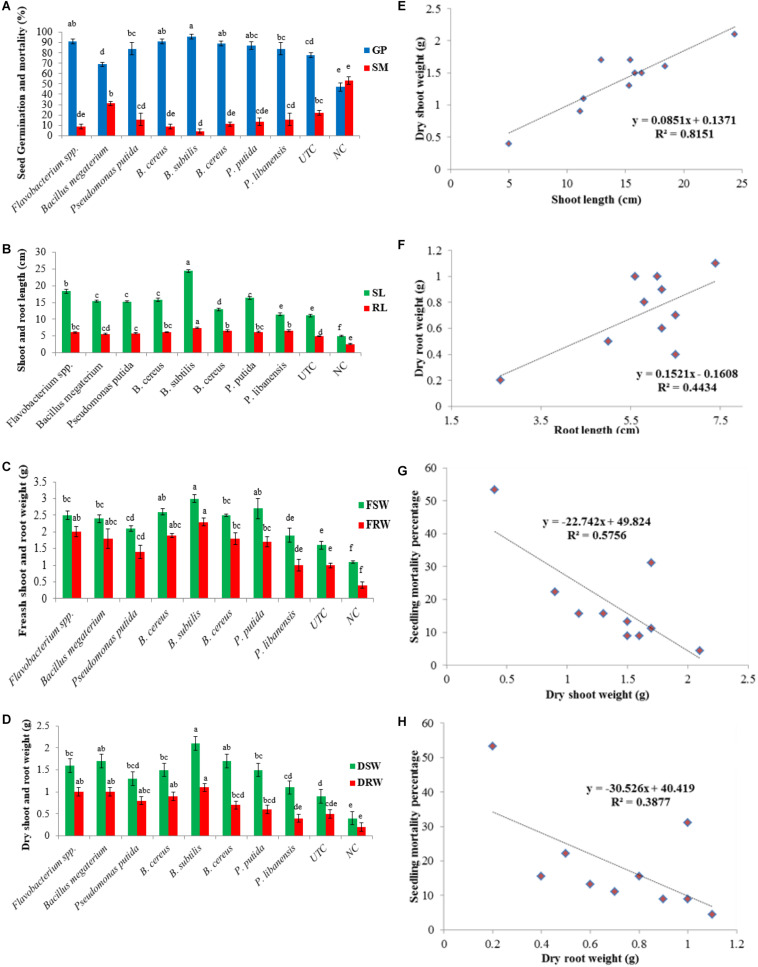
Evaluation of rhizobacteria isolated from chili growing fields for the suppression of *Pythium myriotylum* and plant growth promotion effects on chili seeds in pot trials. Zoospores of *P. myriotylum* were added into the soil and bacterized chili seedlings were sown in sick soil containing pots in three repeats. Error bars show standard deviations and letters on each bar represent the significant difference among the values at 5% level of significance. **(A)** seed germination and seed mortality percentage, **(B)** shoot and root length, **(C)** fresh shoot and root weight, **(D)** dry shoot and root weight, **(E)** positive correlation between shoot length and dry shoot weight, **(F)** positive correlation between root length and dry root weight, **(G)** negative correlation between dry shoot weight and seedling mortality percentage, **(H)** negative correlation between dry root weight and seedling mortality percentage.

Considering the biocontrol and plant growth promotion traits, rhizobacterial isolates were screened for disease suppression and plant growth promotion in chili plants under an open environment, and data on disease suppression, seed germination, and plant growth traits were recorded. All the tested bacterial isolates significantly improved seed germination in the presence of pathogenic fungi in pot soil. Maximum seed germination of 96% was produced by *Bacillus subtilis* followed by *Flavobacterium* spp. (91%) and *B. cereus* (89%) while *B. megaterium* showed the least effect on seed germination (70%) over the negative control – NC (47%). All the tested bacterial isolates suppressed the pathogenic fungi and significantly lower the seedling mortality ranging between 4.4 and 31% as compared to negative control where seedling mortality was recorded 53% (Figure 7A). A significant increase in shoot length (*P* ≤ 0.05) was seen in all the treatments of bacterial isolates. A significant increase in shoot length (24.4 cm/plant) was observed in pots treated with *B. subtilis* followed by *Flavobacterium* spp. (18.4 cm/plant) over untreated control treatment-UTC (11.1 cm/plant) and negative control-NC (5 cm/plant). All the bacterial antagonists significantly enhanced the root length ranging 5.6–7.4 cm/plant over untreated control - UTC (5 cm/plant) and negative control - NC where the root length was recorded 2.6 cm/plant (Figure 7B). An increase in fresh shoot weight (*P* ≤ 0.05) was observed in all the pots treated with bacteria isolates and an increase in fresh shoot weight ranged from 1.8 to 3.0 g compared to UTC (1.6 g) and NC (1.1 g). Data recorded from all the bacterial treated pots showed that the increase in the fresh root weight ranged between 0.96 and 2.3 g compared to un-inoculated pots (0.93 g) and the negative control (0.4 g), as given in Figure 7C. A significant increase in dry shoot weight (*P* ≤ 0.05) was also ranged from 1.1 to 2.1 g over un-inoculated pots (0.93 g) and negative control (0.4 g). Among all the tested isolates *B. subtilis* showed the highest increase in dry shoot weight (2.1 g) while *P. libanensis* showed the least significant increase in dry shoot weight (1.1 g). Data on dry root weight displayed that all the tested bacteria isolates had significantly increased the dry root weight (*P* ≤ 0.05) in chili plants ranging from 0.36 to 1.13 g over the untreated control (0.46 g) and negative control (0.16 g) (Figure 7D). A positive correlation was observed between the shoot, root length, and dry shoot, root weight (Figures 7E,F) whereas a negative correlation was recorded between dry shoot, root weight, and chili seedling mortality percentage (Figures 7G,H).

## Discussion

The damping-off of chili pepper by *Pythium* spp. causes decay of germinating seeds and growing seedlings at pre and post-emergence growth stages, and represents the huge yield constraints faced in both nurseries and field conditions (Erwin and Ribeiro, 1996). The estimated yield loss due to this disease could be from 5 to 80% under favorable conditions (Lamichhane et al., 2017). In our study, young symptomatic chili seedlings (15 to 30 days old) showed the damping-off, reduced growth, wilting, water soaked lesions, brown discoloration, and root rot due to *Pythium* infection. These symptoms confirm the findings of Horst (2013). A total of 13 *Pythium* isolates were recovered from these infections and were purified onto corn meal agar (CMA) medium amended with ampicillin (250 mg/L), rifampicin (10 mg/L), and pimaricin (10 mg/L) (Jeffers and Martin, 1986). The morphological features of these isolates including; coenocytic hyphae with lobate sporangia, knob-like appressorium, zoospores diameter, oogonia diameter, antheridia, and aplerotic oospores of the isolates fit well with descriptions of *Pythium myriotylum* Drechsler described in previous literature (Drechsler, 1943).

Seven day old fungal cultures were transferred to CMA medium for zoospore production by following the methodology previously described by Rahimian and Banihashemi (1979). The inoculum artificially applied to healthy plants under greenhouse conditions produced symptoms of damping-off and root rot compared to control plants that remained asymptomatic. This confirmed Koch’s theory. The virulence of *P. myriotylum* has been reported in various studies (Kageyama and Ui, 1983; Okada, 2003; Tomioka et al., 2013). In another study, the pathogenicity of *P. aphanidermatum* was confirmed in tomato and chili seedlings by Ramamoorthy et al. (2002a). The two most aggressive isolates, PMyr-1 and PMyr-2, in pathogenicity trials were subjected to molecular characterization by amplifying the ITS1 and ITS2 regions as described by White et al. (1990). The amplified sequences of MF143429 and MF143430 (approximately 700-bp) showed 99% sequence homology with HQ643704 accession of *P. myriotylum* previously described by Robideau et al. (2011). Similar results on molecular characterization of *Pythium* spp. were reported by Tomioka et al. (2013).

Among various disease management practices, chemical seed treatment has been adopted extensively (Rothrock et al., 2012; Kandel et al., 2016), and a variety of chemicals have been used as seed dressers to remove the pathogens from the seeds (Mancini and Romanazzi, 2014). However, chemical seed treatment adversely affects seed germination and can cause phytotoxicity (du Toit, 2004). Moreover, the non-judicial application of synthetic chemicals is a potential threat to human health and the environment (Ouyang et al., 2016), and is noxious to the beneficial rhizosphere microbes (Hussain et al., 2009), also resulting in the development of resistance in pests (Onstad, 2013), and increasing public security concerns in many countries (Bourguet and Guillemaud, 2016). Many of these chemicals are declared a carcinogen in a number of countries (Bressa et al., 1997). To minimize dependency on synthetic agrochemicals, scientists have devised alternative eco-friendly approaches to managing crop diseases more sustainably. Previous studies have highlighted the successful application of plant growth promoting rhizobacteria (PGPR) as an alternative to synthetic agrochemicals (Goudjal et al., 2014; Suwitchayanon et al., 2018; Miljaković et al., 2020).

The use of PGPR in disease suppression and plant growth promotion (PGP) is a widely adopted strategy in various crops such as wheat (Abbasi et al., 2011), rice (Yasmin et al., 2016), okra (Begum et al., 2012), cucumber (Islam et al., 2016), sweet pepper (Sid et al., 2003), red pepper (Lim, 2010), avocado (Cazorla et al., 2007), potato (Kenawy et al., 2019), and tomato (Szczech and Shoda, 2004). Souza et al. (2015) reported rhizobacteria belonging to *Pseudomonas*, *Azospirillum*, *Azotobacter*, *Klebsiella*, *Enterobacter*, *Alcaligenes*, *Arthobacter*, *Burkholderia*, *Bacillus*, and *Serratia* spp. enhance plant growth, and are being used as bio-controls (Labuschagne et al., 2010; El-Sayed et al., 2014; Ganapathy and Natesan, 2018).

In the present study, rhizobacterial isolates were screened for the biocontrol of damping-off and plant growth promotion traits in chili crops. Out of the 110 rhizobacterial isolates, 28 (22.7%) showed varied levels of antagonistic potential. Out of these 28 bacterial isolates, eight (28.6%) isolates *Flavobacterium* spp., *B. megaterium*, *P. putida*, *B. cereus*, *B. subtilis*, *B. cereus*, *P. putida*, and *P. libanensis* showed significantly high antagonistic potential against two highly virulent strains of *P. myriotylum* (PMyr-1 and PMyr-2).

The biological control of *Pythium* spp. could be due to the antagonistic ability of the rhizobacteria (Clark, 2006; Elazzazy et al., 2012). This antagonistic potential is due to antibiosis, as various antibiotics have been identified and reported in previous studies (Raaijmakers et al., 2002; Nielsen and Sørensen, 2003). The production of antibiotics and lytic enzymes damage the fungal cell membrane and inhibits the zoospores of Oomycetes (Beneduzi et al., 2012).

Bacterial isolates showing the highest antagonistic potential were subjected to biochemical featuring. Out of the eight bacterial isolates, four were gram positive and others were gram negative. Bacterial isolates belonging to *Bacillus* and *Flavobacterium* spp. showed negative results for fluorescence emission test, while those belonging to *Pseudomonas* spp. respond positively. For the potassium hydroxide (KOH) test, bacterial isolates *Flavobacterium* spp., *P. putida* and *P. libanensis* showed positive test results while other isolates were negative for the KOH test. All the tested bacterial isolates were catalase positive. Many studies have highlighted the catalase production by rhizobacteria (Ali Kamboh et al., 2009; Kumari et al., 2018). Catalase positive bacteria were reported to suppress early blight disease in tomatoes (Senthilraja et al., 2013) and induced resistance against tomato yellow leaf curl virus (Li et al., 2016). Out of the eight bacterial isolates, *B. cereus* and *P. libanensis* were positive for levan production while *P. putida* and *B. cereus* were fermenting the carbohydrates. With the exception of *P. libanensis*, all the bacterial isolates exhibited positive responses for the oxidase test except *B. subtilis*. All the tested bacterial isolates showed nitrate reduction, except for *Flavobacterium* spp. and *P. libanensis*. All the tested bacterial isolates showed gelatin hydrolysis activity except for *Flavobacterium* spp. In a similar study, rhizobacterial strains were differentiated based on morphology and biochemical traits (Singh et al., 2019).

The 16S rRNA sequences have been widely used in the classification and identification of Bacteria and Archaea (Goodfellow et al., 2014). The sequence analysis of bacterial isolates indicated that the tested bacterial isolates belonged to three different genera including *Flavobacterium* spp., *Bacillus* spp. and *Pseudomonas* spp. In our studies, 16S rRNA sequence-based neighbor-Joining (N-J) tree indicated that two bacterial isolates ID (MH393211) and 1C2 (MH393210) showed 97 to 98% sequence identity with *Bacillus cereus* (accessions MK606105 and MK648339) while JHL-8 (MH393209) showed 99% sequence homology with *B. megaterium* (accession MG430236). The sequence of RH-24 (MH393208) had 99% sequence homology with *B. subtilis* (accession KY000519) and RH-87 (accession MT421780) showed 99% identity with *Pseudomonas libanensis* (DQ095905). Two bacterial isolates 5C (MH371201) and JHL-12 (MH371200) had 99% sequence homology with *P. putida* (accessions KY982927 and MF276642, respectively). Bacterial isolate 4a2 (accession MT421823) had 99% identity with the *Flavobacterium* spp. (accession HM745136). Similar 16S rRNA gene sequence based rhizobacterial characterizations have been reported in other literature (Islam et al., 2016; Kuan et al., 2016; Kumari et al., 2018; Zouaoui et al., 2019).

Bacterial strains were also tested for plant growth promotion traits. All the antagonistic bacterial produced ammonia (NH_3_). Ammonia production is the most common character of PGPR, which indirectly enhances plant growth (Yadav et al., 2010). It accumulates and supplies nitrogen to the host plants and helps in plant growth promotion (Kumar et al., 2016), and it also contributes to antagonism (Howell, 1988). Various researchers have cited the production of ammonia by rhizobacteria (Jayasinghearachchi and Seneviratne, 2004; Triveni et al., 2012; Mazumdar et al., 2019). Furthermore, all the tested bacterial isolates except *Flavobacterium* spp. were able to hydrolyze starch. The production of HCN by PGPR is independent of their genus. Except for *B. cereus* and *Flavobacterium* spp., all the bacterial strains were positive for the HCN production test. Previous researches have documented that the bacterial agents with HCN producing ability can be used as biocontrol agents (Ramette et al., 2003). It is now believed that HCN production indirectly increases phosphorus availability by chelation and sequestration of metals and indirectly increases the nutrient availability to the rhizobacteria and host plants (Rijavec and Lapanje, 2016), and they are thus used as biofertilizers or as a bio-control to enhance crop production (Agbodjato et al., 2015).

All the bacterial strains were also evaluated for P-solubilization, indole-3-acetic acid (IAA), and siderophore production. Phosphate solubilization is an important plant growth promotion trait of rhizobacteria; in which rhizobacteria produce low molecular weight organic acids which solubilize phosphate thus, lowering the pH of the soil (Khan et al., 2014) thus, converts the phosphate into available forms that is taken up by the plant roots (Ahmad et al., 2018). In this study, all the bacterial isolates belonging to *Flavobacterium*, *Bacillus*, and *Pseudomonas* spp. showed P-solubilization ability, and the test was not performed for *B. cereus*. Indole-3-acetic acid (IAA) is a vital phytohormone that is involved in root development, elongation, proliferation and facilitates plants to obtain water and nutrients from the soil (Yao et al., 2008). It increases the root surface area and loosens the plant cell walls, which facilitates in getting soil nutrients and supports better plant microbe interaction (Glick, 2012). In our study, all the bacterial isolates produced a considerable amount of IAA (13.4–39.0 μgmL^–1^) which were comparable to previously published reports by Islam et al. (2016) and Zahid et al. (2015). The increase in shoot and root length in bacterial inoculated plants may be attributed to their ability to produce IAA. Siderophore production is one of the most influential traits exhibited by plant growth promoting rhizobacteria especially when iron availability is limited (Whipps, 2001) and suppress the phytopathogens by depriving them of available iron (O’sullivan and O’Gara, 1992). All the PGPR tested in this study showed promising siderophore production (17.1–27.7%) which proves rhizobacterial ability to suppress the growth of target pathogen *P. myriotylum*. Many previous reports have supported the siderophore production potential of PGPR and its role in disease suppression (Swadling and Jeffries, 1996; Compant et al., 2005; Sayyed et al., 2019; Ali et al., 2020; Kesharwani and Singh, 2020; Sharf et al., 2021).

Previous studies have proven that heavy metal ions co-regulate genes that confer antibiotic resistance and decrease antibiotic susceptibility (Baker-Austin et al., 2006; Rani et al., 2010). In this study, bacterial isolates were screened for their resistance or susceptibility response against Streptomyces, ampicillin, rifampicin, penicillin G, and vancomycin at five dose levels, and all the tested bacterial strains displayed a varied level of resistance and antibiotic susceptibility response. The study has shown that the tested bacterial isolates have varied levels of tendencies to overcome the antibiotics stress and it might be associated with tolerance against heavy metals present in the soil. Bacterial isolates showing multiple antibiotic resistances have greater chances to establish as inoculum in the soil and any new niche. Metal tolerance and antibiotic resistance have previously been reported in many studies (Thacker et al., 2007; Wani and Irene, 2014).

Treatment of chili seed with selected bacterial strains significantly improved seed germination, plumule and radical length, and vigor index as compared to un-inoculated control. No phytotoxicity and stress on seedling germination had been observed in any treatment. All the antagonists used in this study were non-pathogenic to chili seeds. As these bacterial isolates showed P-solubilization and IAA activity thus, can be used for plant growth promotion (Naureen et al., 2009) and the growth enhancement may be due to the production of IAA. A research study has shown high amylase activity during seed germination in rice and legume inoculated with PGPR (Duarah et al., 2011). A recent study reported that seed treatment with PGPR significantly improved seed germination, plant growth promotion, fresh weight, and improved root formation in green-gram and maize crops (Oo et al., 2020).

The level of defense-related enzymes contributes significantly to the mechanism of host plant resistance (Shivakumar et al., 2000). The bacterial inoculated chili plants also displayed a significant increase in defense related enzyme activities. Chili seedlings treated with bacterial isolates exhibited a significant increase in Peroxidase (PO), Polyphenol oxidase (PPO), and Phenylalanine ammonialyas (PAL) activities. PO activity was maximum in the seedlings treated with *Flavobacterium* spp. followed by *B. megaterium* and *B. subtilis* while PPO and PAL activity was recorded significantly high 5 days after inoculation (DAI) in chili roots bacterized with *B. subtilis* inoculum over un-inoculated (UC) and negative control (NC). Our results are supported by the findings of Benhamou and Paulitz (2000) where cucumber roots inoculation with *Pseudomonas corrugate* and *P. aureofaciens* suppressed the root and crown rot caused by *P. aphanidermatum* and PAL accumulation lasted for 16 days while Peroxidase (PO) and Polyphenol oxidase (PPO) activities were enhanced in roots 2 to 5 days after bacterial treatment. It was previously reported that the induction of plant defense related enzymes is related to the plant defense system and induced resistance by PGPR inoculation and colonization (Liang et al., 2011). The accumulation of defense related enzymes after PGPR application has also been reported in cucumber (Liang et al., 2011), chili (Jayapala et al., 2019), and tomato (Ramamoorthy et al., 2002b). Dukare and Paul (2021) also found that rhizobacterial inoculation conferred resistance to pigeon pea seedlings by inducing and improving the production of defense related enzymes and phenolics.

Many studies have proved that PGPR has great potential to suppress plant pathogens and increase plant growth under greenhouse conditions (Kabdwal et al., 2019). These PGPR induce systemic resistance against a broad spectrum of pathogens due to their root colonization ability (Salem and Abd El-Shafea, 2018). Both *Pseudomonas* and *Bacillus* spp. are known for their role in disease suppression against various plant pathogens (Velusamy et al., 2013). In present studies, tested bacterial strains enhanced the chili germination percentage and reduced the seedling mortality percentage by suppressing the *P. myriotylum*, and a significant increase in plant growth characters was observed in bacterial inoculated treatments over un-treated (UTC) and negative control (NC). Similar results have been reported in other research studies (Egamberdieva, 2011; Almaghrabi et al., 2013; Islam et al., 2016; Torres et al., 2020). Athira and Anith (2020) reported that tomato seed treatment with *R. radiobacter*, *S. leeuwenhoekii* and *P. indica* significantly minimized the disease incidence of bacterial wilt disease of tomato.

The biocontrol of plant pathogens is not very popular in field conditions as the crop is more open to a range of pests that may prevent the development of a specific biological control. Climatic conditions and soil factors also influence the potential of biocontrol agents against disease suppression. Many studies have discussed the low performance of bacterial-based products under open field conditions due to various climatic and soil factors (Ownley et al., 2003), which affect bacterial colonization ability, biological activates, and disease suppressing potential (Landa et al., 2001). Biocontrol is a complex phenomenon involving several mechanisms in disease suppression and understanding these mechanisms would be beneficial for the effective utilization of bacterial biocontrol agents in open fields. Many biocontrol products against damping-off disease are available worldwide (Lamichhane et al., 2017) but not a single locally prepared product is available and registered in Pakistan. Bio-products imported from other countries failed to perfume due to the nature of different soils and climatic conditions. Taking this into account, this study aimed to screen out the native bacterial antagonists with high disease suppressive and plant growth promotion abilities. To our knowledge, the antagonistic potential of native PGPR was first reported in Pakistan. However, a series of further experiments are required to test the efficacy of these bacterial isolates at different dose levels and formulations with different soil types and climatic conditions. Finally, field trials will help to develop the bacterial based bioproduct, its registration, and commercialization.

## Conclusion

Plant disease suppression and growth promotion are considerable aspects in achieving good quality produce. Chili pepper is cultivated as an important vegetable crop across the world, and its production is greatly reduced by the damping-off disease caused by *Pythium myriotylum.* In response, this study screened rhizobacterial isolates *in vitro* which showed greater potential to suppress *P. myriotylum* inoculum, and significantly improved the seedling germination and vigor index without posing any phytotoxicity and pathogenic impact on young seedlings. These bacterial isolates showed P-solubilization, indole-3-acetic acid, and siderophores production ability and produced varying levels of susceptibility and tolerance response against antibiotics. The application of bacterial isolates increased the accumulation and activities of defense related enzymes (PO, PPO, and PAL) in chili roots under pathogen pressure. These PGPR, which have multiple disease suppressive and PGP traits, significantly reduced the seedling mortality and improved the seedling germination and other PGP traits in pot trials. However, more detailed study is required, examining dose calibration, and field performance testing of these PGPR is required to ensure the safe application of these bacteria as biocontrol agents.

## Data Availability Statement

The data supporting the findings of this study are available within the article.

## Author Contributions

SH designed research, conducted experiments, and wrote manuscript. AG helped in conducting research experiments, data analysis, manuscript write up, and proofreading. ZR helped in data analysis and proofreading. RA helped in data analysis and interpreting the results. MISH and NF helped in research work and data collection. MI conceived the idea and supervised the research work. All authors contributed to the article and approved the submitted version.

## Conflict of Interest

The authors declare that the research was conducted in the absence of any commercial or financial relationships that could be construed as a potential conflict of interest.

## Footnotes

1 https://blast.ncbi.nlm.nih.gov
